# Resident CD8^+^ and Migratory CD103^+^ Dendritic Cells Control CD8 T Cell Immunity during Acute Influenza Infection

**DOI:** 10.1371/journal.pone.0066136

**Published:** 2013-06-04

**Authors:** Jason Waithman, Damien Zanker, Kun Xiao, Sara Oveissi, Ben Wylie, Royce Ng, Lars Tögel, Weisan Chen

**Affiliations:** 1 Telethon Institute for Child Health Research and Centre for Child Health Research, University of Western Australia, Perth, Western Australia, Australia; 2 Ludwig Institute for Cancer Research (Melbourne-Austin Branch), Melbourne, Victoria, Australia; 3 School of Molecular Science, La Trobe University, Melbourne, Victoria, Australia; Massachusetts General Hospital, United States of America

## Abstract

The identification of the specific DC subsets providing a critical role in presenting influenza antigens to naïve T cell precursors remains contentious and under considerable debate. Here we show that CD8^+^ T lymphocyte (T_CD8+_) responses are severely hampered in C57BL/6 mice deficient in the transcription factor Batf3 after intranasal challenge with influenza A virus (IAV). This transcription factor is required for the development of lymph node resident CD8^+^ and migratory CD103^+^CD11b^−^ DCs and we found both of these subtypes could efficiently stimulate anti-IAV T_CD8+_. Using a similar *ex vivo* approach, many publications on this subject matter excluded a role for resident, non-migratory CD8^+^ DC. We postulate the differences reported can partially be explained by how DC are phenotyped, namely the use of MHC class II to segregate subtypes. Our results show that resident CD8^+^ DC upregulate this marker during IAV infection and we advise against its use when isolating DC subtypes.

## Introduction

Dendritic cells (DC) are a heterogeneous bone marrow-derived cell population widely accepted as the key initiators of T cell immunity [1]. Considerable complexity surrounds understanding the requirement for such heterogeneity along with pinpointing the underlying role each subset has evolved to provide. DC heterogeneity is most apparent in lymph nodes that drain peripheral sites such as the lung, skin and gastrointestinal tract. These lymph nodes are comprised of two major subsets: plasmacytoid DC and myeloid DC, with the latter commonly referred to as conventional or classical DC (cDC) [2].

cDC isolated from the lymph node that drains the lung represent a mixture of lymphoid resident DC, which do not traffic from the periphery, and tissue-derived, “migratory” DC. Lymphoid resident DC subsets can be subdivided into CD8α-expressing DC (CD8^+^ DC) and CD8^−^ DC [3], [4]. Two defined migratory DC subsets exist and are subcategorized based on their differential expression of the mucosal α_E_ integrin marker CD103 and myeloid marker CD11b (CD103^+^CD11b^−^ DC and CD103^−^CD11b^+^ DC) [5].

Currently, considerable contention surrounds immune control by these subsets during acute influenza infection [6], [7], [8], [9], [10], [11], [12], [13]. Evidence exists for either anti-IAV T_CD8+_-mediated immunity elicited by a combination of lymph node resident and migratory DCs or alternatively sole control by both or only one of the migratory DC populations. The vast majority of these studies utilize *ex vivo* DC analysis, which is reliant on astute segregation of distinct subsets based on lineage markers defining end-stage subpopulations. In addition, pan-DC markers are widely used; these include surface expression of CD11c and Major Histocompatibility Class II molecules (MHC II). The expression level of MHC II on cDCs is variable and dependent on maturation status. In the absence of infection, cDCs in the lung parenchyma and lymphoid-resident DC in lymphoid organs are present in a so-called “immature state” dedicated to surveillance, not T cell priming. In such a state, they possess a high endocytic/phagocytic capacity and intermediate levels of surface MHC class II (MHC II^int^). Tissue-derived DC constitutively traffic to the lymph nodes and terminally differentiate into mature cells dedicated to modulating T cell immunity. The coordinated phenotypic changes associated with this maturation event include downregulation of endocytic/phagocytic activity, upregulation of costimulatory molecules and increased surface expression of antigen-MHC II complexes (MHC II^high^) [14].

These basic life-cycle events are often extrapolated to conditions when inflammatory stimuli, such as influenza infection, are present. Multiple groups segregate migratory DC and lymphoid resident DC into MHC II^high^ and MHC II^int^ populations, respectively, from the draining mediastinal lymph nodes of influenza infected animals. While DC maturation during acute influenza infection is reported, notably upregulation of the costimulatory molecule CD86 [7], [9], altered expression of MHC II in DC subsets has not been thoroughly characterized.

Here we report mice deficient for the basic leucin zipper transcription factor 3 (Batf3^o/o^) [15], [16], which have a selective deficit in CD8^+^ and CD103^+^CD11b^−^ DC within the mediastinal lymph node, lack anti-IAV T_CD8+_ immunity. This result implicates a crucial role for one or both of these subsets in orchestrating T_CD8+_ immunity during an acute viral infection. Our study shows that both *ex vivo-*isolated lymphoid resident CD8^+^ and migratory CD103^+^CD11b^−^ DC are capable of MHC class I-restricted presentation after lung infection. Multiple studies incorporate a similar *ex vivo* approach to interrogate DC subset contribution for generating T_CD8+_ immunity and do not reach the same conclusion [17]. This prompted us to further scrutinize the phenotypic changes associated with DC behavior during the early stages of IAV infection. Our results show that the lymphoid resident CD8^+^ DC population rapidly upregulates MHC II complexes early during infection. Multiple studies employ this marker to distinguish between resident and migratory DC. Our results suggest not relying on this marker when isolating DC subsets, especially during an inflammatory setting. This is an important point as it identifies a potential experimental pitfall and we hope it provides the first steps to providing an explanation into the outstanding discrepancies that exist within the current literature. This question is something that has puzzled the field for a long time and it is paramount that a unified view on this matter be reached in order to move the research forward.

## Materials and Methods

### Mice and virus infections

Female B6.Ly5.1 and B6.Ly5.2 (H-2^b^) mice were purchased from the Walter and Eliza Hall Institute. B6 Batf3^o/o^ were provided by K.M. Murphy [15] and maintained at either the Ludwig Institute for Cancer Research or Telethon Institute for Child Health Research. Mice were inoculated intranasally with 100 pfu of A/Puerto Rico/8/34 (PR8). All animal experiments were performed in accordance with protocols approved by both the Austin Health Animal Ethics Committee (Ethics Application ID: 02983) and the Telethon Institute for Child Health Research Animal Ethics Committee (Ethics Application ID: 243) and conformed to the National Health and Medical Research Council Australia code of practice for the care and use of animals for scientific purposes.

### Fluorescent labeling of DC migration in the lung

PKH26 dye was diluted in diluent C (Sigma) 1∶100 and 40 µL was administered by intranasal delivery to each mouse after anesthesia.

### Generation of T_CD8+_ line specific to IAV NP_366–374_

Splenocytes were isolated from B6.Ly5.1 mice primed with PR8 at least 30 days prior to harvest. 10% of these cells were pulsed with 1 nM NP_366–374_ for 60 minutes. The cells were then washed with PBS and co-cultured with the remaining 90% of memory splenocytes in the presence of IL-2 (Chiron). T cell lines specific to IAV NP_366–374_ were generated using a recently described protocol [18]. T cell lines showing >90% T cell activation by bone-marrow-derived DC pulsed with NP_366–374_ were used.

### Peptides and intracellular cytokine staining

**Figure 1:**
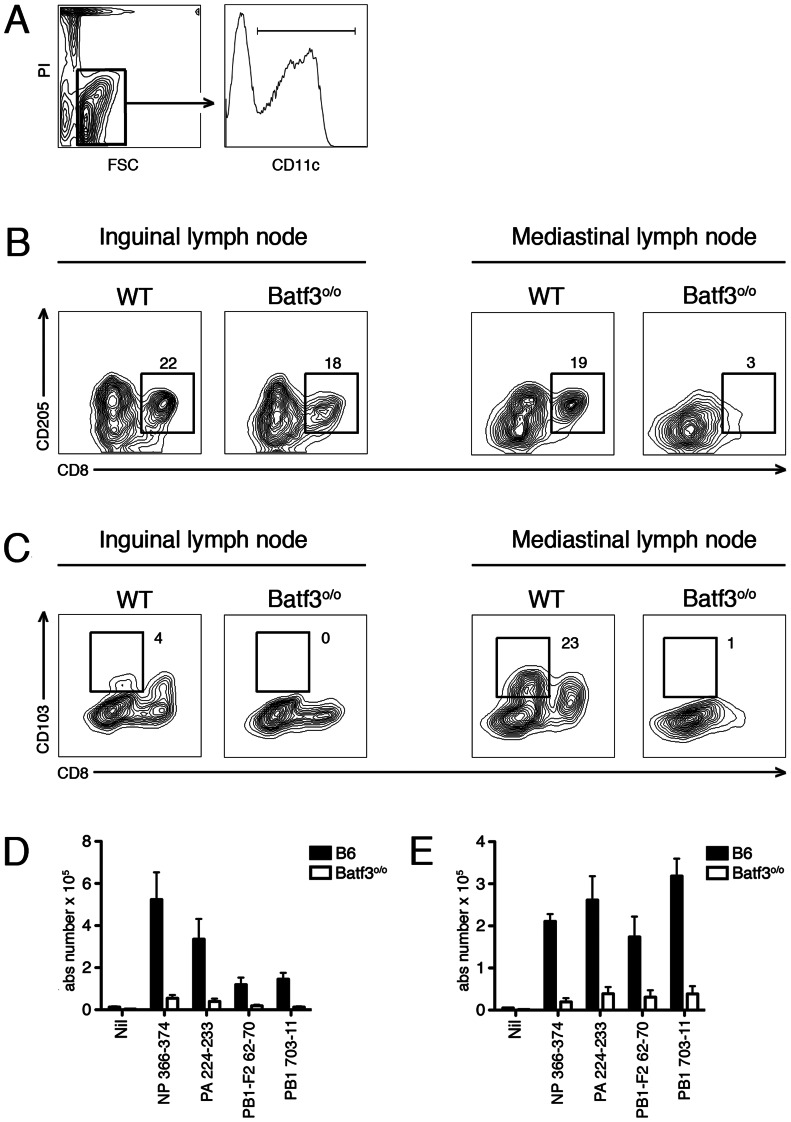
Lack of influenza specific T_CD8+_ responses in Batf3^o/o^ mice. (A) Pre-gating strategy to identify DC (B–C) DC from the inguinal and mediastinal lymph nodes of wildtype or Batf3^o/o^ mice on a B6 background were analyzed for their expression of either (B) CD8 and CD205 or (C) CD8 and CD103. Representative plots from 3-pooled mice from two independent experiments are shown. (D–E) B6 and Batf3^o/o^ mice were infected with PR8. On day 10, the absolute number of influenza specific T cells specific for defined peptide sequences were measured in the spleen (D) or BAL (E). The specific T cell response was elucidated following stimulation without peptides (Nil) or the peptides NP_366–374_, PA_224–233_, PB1-F2_62–70_, or PB1_703–711_. Shown is the absolute number of IFNγ^+^ CD8^+^ T cells, calculated using the following equation: cell count x%PI^−^ x%CD8^+^ x%IFNγ^+^. Average is taken from between 5–6 mice per group over two independent experiments and the error shows the SEM.

**Figure 2:**
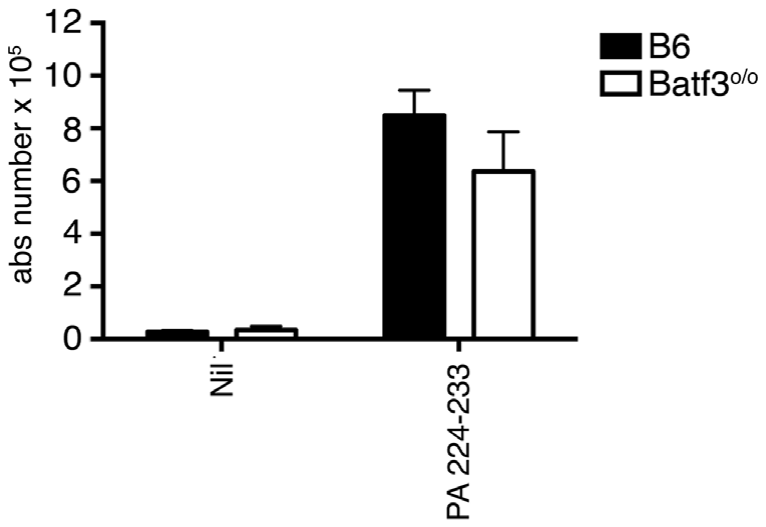
PA_224–33_ T_CD8+_ can be generated in Batf3^o/o^ mice. B6 or Batf3^o/o^ mice were inoculated intraperitoneally with 2.5×10^6^ LPS-treated, PA_224–233_ peptide-pulsed B6 bone-marrow-derived DC. 7 days later, the number of PA_224–233_ responding T_CD8+_ present in the spleen was determined by ICS. Average is taken from 6 mice per group over two independent experiments and the error shows the SEM.

T_CD8+_ responses were enumerated using intracellular cytokine staining (ICS) after being stimulated with 1 μM of the following antigenic peptides: IAV NP_366–374_ (ASNENMETM), PA_224–233_ (SSLENFRAYV), PB1F2_62–70_ (LSLRNPIKV) and PB1_703–711_ (SSYRRPVGI) in the presence of brefeldin A (Sigma). Anti-CD8 and -IFNγ antibodies (BD) were used to identify positive cells in Fig 1–2. Flow cytometrically sorted DC subsets from influenza infected B6.Ly5.2 mice were co-cultured with a CD45.1^+^T_CD8+_ cell line specific to IAV NP_366–374_ for six hours in the presence of BFA. Anti-CD45.1 and -CD8 antibodies (BD) were used to identify the T_CD8+_ line and anti-IFNγ antibody (BD) was used to gauge responsiveness to stimulation.

### DC isolation from lymph nodes and co-culture with IAV-specific T cells

DCs were isolated as previously described [19]. Briefly, single cell suspensions from the mediastinal lymph node were enriched for DC using an antibody depletion and magnetic enrichment. Purity of enriched cell suspensions usually yielded >40% CD11c^+^ DC. These enriched DC were stained with anti-CD11c, -CD8, -CD11b (BD) and -CD103 (eBiosciences) for flow cytometric sorting. Serial dilutions of each sorted DC population were co-cultured *in vitro* with 5×10^4^ NP_366–374_ -specific T cells. Antigen-specific T cells were enumerated by ICS as described above.

### Flow cytometry

Multi-parameter analysis were performed on the FASCcalibur (BD), FASCCanto II (BD) or LSRFortessa (BD) and analyzed with FlowJo software (Tree Star). Monoclonal antibodies specific to mouse CD8α, CD11b, CD11c, CD45.1, CD103 and IFNγ were purchased from either BD or eBiosciences. Anti-MHC II [I-A/I-E] (M5/114), anti-CD86 (GL1), and anti-CD205 (NLDC-205) were made in house. Prior to acquisition, cells were stained with propidium iodide (Sigma) to exclude dead cells.

## Results

### Batf3^o/o^ mice are unable to mount an efficient anti-influenza T_CD8+_ response

DCs are well understood to mediate T cell activation and initiate anti-viral immunity. We previously argued that two specific subtypes, the CD8^+^ and CD103^+^CD11b^–^ DC subsets, drive T_CD8+_ responses during herpes simplex virus skin infection [20]. We sought to determine whether this was also the case during pulmonary IAV infection. We hypothesized that in the absence of the CD8^+^ and CD103^+^CD11b^–^ DC subsets, naïve influenza-specific T cell priming should be compromised. A recent series of novel studies identified that the transcription factor Batf3 is required for the development of both the splenic CD8^+^ and dermal CD103^+^CD11b^–^ DC subset lineages and mice lacking this gene were ideal for testing our hypothesis [15], [16]. However, our experimental models utilize C57BL/6 (B6) mice and mice lacking Batf3 on this background are reported to retain completely normal numbers of functional CD8^+^ DC within the skin-draining lymph nodes [21], [22]. We did observe similar results within the skin-draining inguinal lymph nodes of both B6 wildtype and Batf3^o/o^ mice. Interestingly, a selective loss of the CD8^+^ DC subset remained in the lung-draining mediastinal lymph node of Batf3^o/o^ mice of this background (Fig 1B). Furthermore, a complete loss of the migratory CD103^+^CD11b^−^ DC subset was observed in both skin- and lung-draining lymph nodes (Fig 1C). Thus, Batf3^o/o^ mice on a B6 background remain an excellent opportunity to test our hypothesis.

We infected control B6 and Batf3^o/o^ mice with influenza (strain PR8) and the expansion of various well-characterized influenza-specific T_CD8+_ populations were analyzed at the reported peak of the immune response (10 days p.i.) [23], [24]. Consistent with previous published results, robust numbers of T_CD8+_ specific to NP_366–374_, PA_224–233_, PB1-F2_62–70_, and PB1_703–711_ were observed in both the spleen (Fig 1D) and bronchoalveolar lavage (Fig 1E) of control mice. In contrast, the magnitude of the T cell response to each of these epitopes was clearly diminished in both compartments within Batf3^o/o^ mice (Fig 1D–E). Near identical results were observed following infection with a different virus strain (X-31), further highlighting this impairment in Batf3^o/o^ mice (data not shown). It is important to note that the absence of these DC subsets did not compromise naïve CD8^+^ T cell precursors specific to influenza as robust T_CD8+_ responses against PA_224–233_ were generated in both strains primed with peptide pulsed bone marrow derived-DC (Fig 2). These results provide *in vivo* evidence for a critical role of one or both the CD8^+^ and/or CD103^+^CD11b^−^ DC subsets in initiating T_CD8+_ immunity during acute IAV infection.

### CD8^+^ and CD103^+^ DC are essential for generating optimal anti-influenza T_CD8+_ immunity

**Figure 3:**
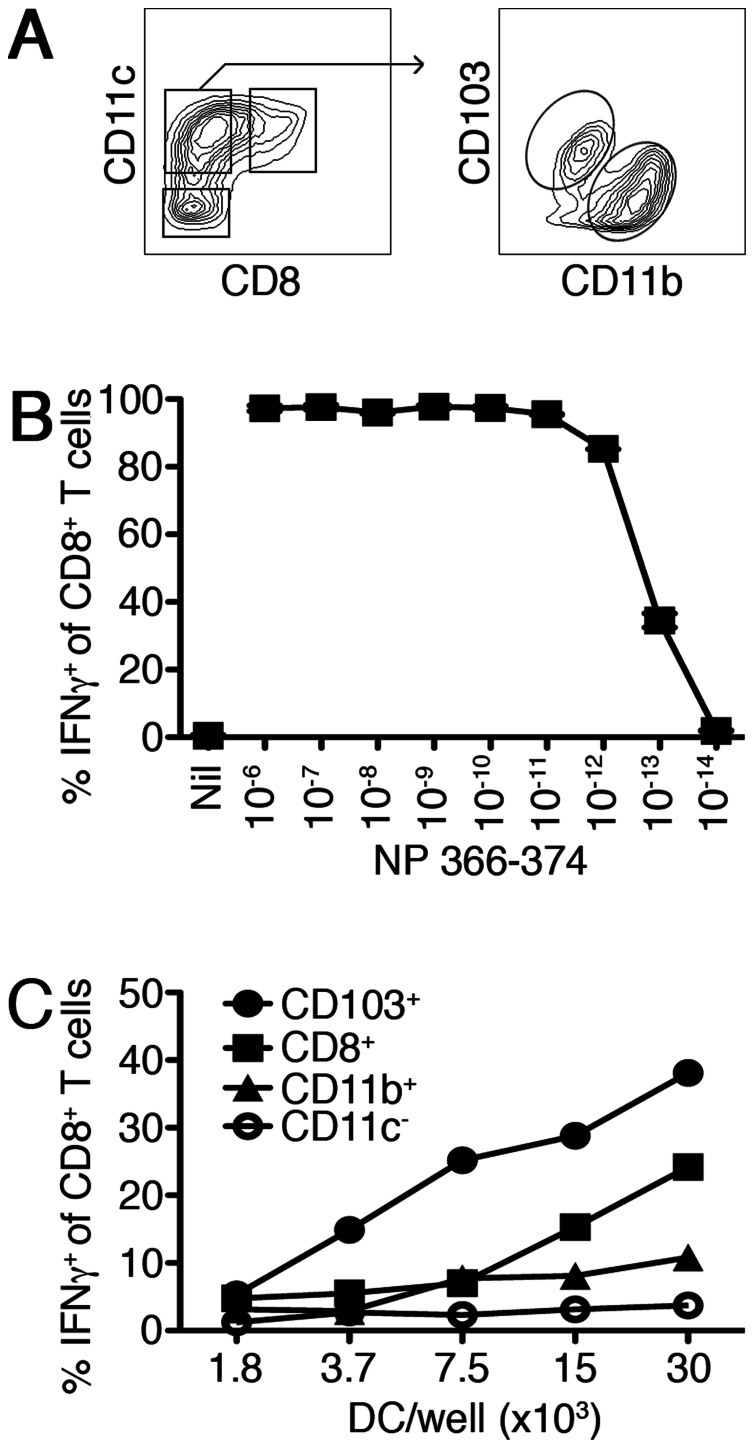
Antigen presentation by DC subsets after influenza infection. B6 mice were inoculated with PR8 and 3 days post infection the lung draining mediastinal lymph nodes were pooled and DC isolated. (A) Gating strategy for isolation of enriched DC subpopulations: CD8^+^ DC were purified on the basis of expression of CD11c and CD8 (upper left; right gate); CD11c^+^CD8^−^ cells (upper left; left gate) were segregated into CD103^+^CD11b^−^ (upper right; top gate) and CD103^−^CD11b^+^ (upper right; lower gate); and finally CD11c^−^ cells were isolated (upper left; bottom gate). (B) The antigen-specific T cell activation for the T cell line specific for the H-2D^b^ restricted influenza epitope NP_366–374_ was assessed using B6 bone-marrow derived DCs pulsed with NP_366–374_ peptide at indicated dilutions in a standard ICS assay for IFNγ. (C) Production of IFNγ by NP_366–374_ T cells (5×10^4^) co-cultured for 6 hours with serially diluted DC subsets as identified in (A). Data are representative of two independent experiments, which showed a similar trend.

We next turned to an *ex vivo* antigenic presentation assay to determine the individual antigen presentation contributions of the different DC subsets, especially those implicated in the above *in vivo* experiments. This involves DC isolation from the lung draining lymph node of IAV-infected mice, segregation into defined subpopulations before co-culture with IAV-specific T cells. If the T cells respond, it is indicative that the co-cultured DC subtype is presenting antigen. We prepared DC at the reported peak of antigen presentation, D3 p.i. [6], by first depleting lymph node suspensions of other cell types and then staining for pan-DC marker CD11c as well as the markers CD8, CD103 and CD11b. Then, we sorted CD11c^+^ DC by flow cytometry on the basis of differences in expression of these markers to delineate the subsets. We collected CD11c^−^ cells as a negative control as well as CD11c^+^CD8^+^ DC before fractionating the CD11c^+^CD8^−^ population into the CD103^+^CD11b^−^ DC subset and a CD11b^+^ subset (Fig 3A). It is imperative to isolate CD8^+^ DC first, as we have previously demonstrated they can express CD103 [20] and could potentially contaminate the migratory CD103^+^CD11b^−^ population. Following sorting, we examined these populations for their presentation of an influenza-derived epitope by co-culturing independent subsets with a T cell line capable of responding to as little as 10^−13^ M of the immunodominant influenza-derived nucleoprotein peptide (NP_366–374_) (Fig 3B). Figure 3C shows that both CD8^+^ and CD103^+^ DC could stimulate NP_366–374_-specific T cells to generate the effector molecule IFNγ. These findings suggest that both these subpopulations have acquired influenza antigen and are capable of evoking anti-IAV T_CD8+_ immunity.

### DC subset maturation during acute influenza infection

**Figure 4:**
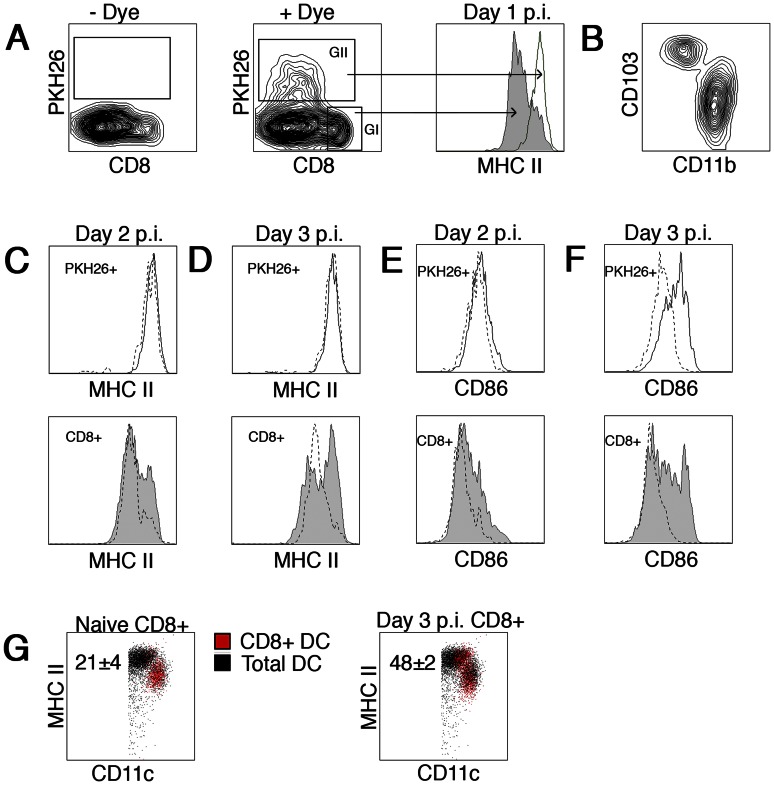
DC subset upregulation of MHC II during acute influenza infection. B6 mice were inoculated i.n. with PKH26 or carrier 16 hours before i.n. infection with influenza virus. One to three days post infection, individual lung draining mediastinal lymph nodes were harvested and DC isolated. Enriched DC were stained with anti-CD11c, anti-CD8, anti-CD103, anti-CD11b, and anti-MHC II and analyzed on a flow cytometer. (A) Representative FACS plots of – Dye (left) and + Dye (middle) treated animals are shown. In addition, a representative histogram overlay from day 1 post infection (right) shows expression of MHC II on the CD11c^+^CD8^+^ DC (Gate I-filled) and PKH26^+^ DC (Gate II-open) subsets. (B) Representative dot plot of pKH26^+^ cells expression of CD103 and CD11b. (C–D) Upper histograms are an overlay showing expression of MHC II on PKH26^+^ cells from naïve (dotted line) or PR8 infected (open) mice day 2 (C) or day 3 (D) post infection. Lower histograms are identical with exception they represent CD8^+^ DC from naïve (dotted line) or PR8 infected (filled) mice. (E–F) Similar to C–D, with the exception expression of CD86 is shown. (G) Dot plot shows CD8^+^ DC (red dots) from naïve (left) or D3 PR8 infected (right) mice as compared to total CD11c^+^ DC from naïve mice. The numbers in each plot represent the frequency of CD8^+^ DC in the MHC II^high^ population. Representative histograms from 1 of 5 mice from two independent experiments are shown.

To examine if phenotypic changes occur in migratory DCs trafficking from the infectious site to the lung draining mediastinal lymph node, mice were instilled i.n. with a solution containing the dye PKH26 16 hours prior to influenza infection. Dye-labeled DCs were readily identifiable in the mediastinal lymph node 1–3 days post infection (Fig 4A). CD11c^+^CD8^+^ DC did not stain for the dye, consistent with the notion they are a non-migratory, lymphoid resident cell population [25] (Fig 4A GI). Dye-labeled cells (Fig 4A GII) were a mixture of the migratory CD103^+^CD11b^−^ DC and CD103^−^CD11b^+^ DC populations previously described [5] (Fig 4B). Consistent with previously published results, a higher proportion of migrated pKH26^+^CD103^−^CD11b^+^ DCs was observed (data not shown). Day 1 p.i., the expression of MHC II surface molecules on CD8^+^ DC were at intermediate levels as compared to a high level of expression on their migratory PKH26^+^ counterparts (Fig 4A histogram). However, a clear shift in MHC II expression within the resident CD8^+^ DC subpopulation was readily observed on day 2 (Fig 4C bottom). Upregulation of this marker by the majority of this DC subset was most evident on day 3 (Fig 4D bottom), a time point previously reported as the peak of antigen presentation [6]. In addition, the kinetics of expression of the co-stimulatory marker CD86 in this DC subset mirrored MHC II expression (Fig 4E–F bottom). No alteration in MHC II expression was observed in the PKH26^+^ migratory populations (Fig 4C–D top) between D1-3 p.i.. Interestingly, no alteration in CD86 expression was observed on pKH26^+^ migratory DCs on D2 p.i. (Fig 4E top). Increased expression of this accessory molecule was only observed D3 p.i. (Fig 4F top) in both the pKH26^+^CD11b^+^CD103^−^ and pKH26^+^CD11b^−^CD103^+^ subpopulations (data not shown). These results clearly show that DC subpopulations are responsive to a peripheral pathogenic encounter, progressing from an immature phenotype to a more mature state.

## Discussion

While the above results are in agreement with several of the aforementioned studies [6], [7], it conflicts with multiple reports where one attributes the CD103^−^CD11b^+^ DC as the major antigen presenting cell [8] and another study that deems the CD103^+^CD11b^−^ DC as the responsible subset [11]. Interestingly, both of these studies employed the same TCR-like monoclonal antibody to detect recombinant IAV-derived H-2K^b^/SIINFEKL [26]. Additional recent studies describe influenza antigen presentation restricted to both CD103^+^CD11b^−^ and CD103^−^CD11b^+^ migratory DC subsets [9], [10]. In one case, the CD103^+^CD11b^−^ DC subset preferentially stimulated naïve T_CD8+_ differentiation [9], while in the second study this was achieved by CD103^−^CD11b^+^ DC [10]. The simple fact so many contradictory results are prominent within the literature clearly identifies a problem. The studies comprehensively rely on *ex vivo* presentation assays, which in turn rely on segregation of DC via lineage markers. It is plausible that the discrepancies may partly be attributed to differing gating strategies prompting an analysis of the various DC populations for phenotypic changes during IAV infection. Our results demonstrate that lymphoid-resident DCs increase their expression of MHC II during a peripheral viral infection, indicating they are transitioning from an immature to a mature phenotype.

These findings draw awareness to gating strategies based on separating lymphoid resident and migratory DC subpopulations using MHC II expression levels in an inflammatory setting. This strategy was utilized in several publications that concluded lymphoid resident DC were not involved in generating anti-influenza T_CD8+_ responses [9], [10]. It is possible that the gating sequence these groups employed place matured lymphoid resident DC, particularly the CD8^+^ subset, into the MHC II^high^ gate. In fact, we readily observe many of the CD8^+^ DC subpopulation undergoing a phenotypic change from CD11c^high^MHC II^int^ in naïve mice to CD11c^int^ MHC II^high^ expression in IAV infected mice (Fig. 4G red dots). This latter phenotype is a classical migratory DC phenotype and we believe a segregation strategy based on MHC II and CD11c expression would ultimately misrepresent both lymphoid resident and migratory DC subsets by excluding a large proportion of the matured resident DC and placing them into the migratory subsets as a contaminating population. Such an experimental design possibly provides misinformation on the identity of the DC groups capable of providing T cell stimulation. In fact, a large proportion of the influenza-induced matured CD8^+^ DC (Fig 4) would be completely overlooked and a false negative result could be observed when the remaining MHC^high^-depleted CD8^+^ subpopulation is utilized in *ex vivo* experiments.

While our results reiterate a crucial role for CD8^+^ and CD103^+^CD11b^−^ DC subsets during IAV infection, it is difficult to ascribe overall individual contributions by either subset from these experiments. Our experiments displayed a trend of less efficient antigen presentation by the CD8^+^ DC subpopulation as compared to CD103^+^CD11b^−^ DC subpopulation (Fig 3C). From our data, it is difficult to interpret if this is on a per cell basis or if far fewer CD8^+^ DC are involved in antigen presentation. However, clearly both populations possess influenza antigens; therefore, retaining the capacity to stimulate naïve influenza-specific precursors recruited to the draining lymph node. Each may further provide unique independent roles, such as the CD103^+^CD11b^−^ migratory DC acting as a transporter to donate antigenic cargo to the CD8^+^ DC in order to amplify T_CD8+_ immunity [25]. It is well documented that the CD8^+^ DC subset excels at cross-priming T cells and in a recent study Langlois et al. imply cross-presentation is a significant pathway used to generate influenza specific T_CD8+_ immunity [27]. Furthermore, we recently published both subsets possess the capacity to prime herpes simplex virus specific T cells and that the dermal CD103^+^CD11b^−^ DC subset specializes in cross-presentation [20]. Thus, we believe a central role for these DC in providing anti-viral immunity, with significant contribution from the cross-presentation pathway, is becoming increasingly apparent. Multiple groups report CD11b^+^ DC play a key role in promoting influenza-specific T_CD8+_ immunity. If this is the case, they evolved to rely on factors supplied by the CD8^+^ and CD103^+^CD11b^−^ DC subsets.

In summary, the role of DC subsets following influenza infection of the lung continues to be an active and intense area of debate. Our results directly challenge recent proposals and provide strong support to the original descriptions of DC presentation of antigen after influenza virus infection. Considering the complex network of DC subtypes require careful and elegant techniques for their identification and isolation, we suggest caution by DC biologists when incorporating MHC II as a segregation marker for *ex vivo* analysis of DC under inflammatory settings such as influenza infection. Finally, we hope this study provides the first steps in unifying the view in the field on specific DC subpopulation control of T_CD8+_ responses during IAV infection and that it provides an explanation for some of the discrepancies. Knowledge of the specific DCs orchestrating anti-viral immunity is critical for the development of targeted vaccination strategies that will provide broad-spectrum T_CD8+_ immunity against IAV.
